# Development and validation of a nomogram prediction model for factors influencing ^131^I-refractory Graves’ hyperthyroidism

**DOI:** 10.3389/fendo.2025.1628226

**Published:** 2025-08-13

**Authors:** Kehua Liao, Xiaojuan Wei, Yan Chen, Dongyun Meng, Shaozhou Mo, Zeyong Sun, Fengyang Song, Lu Lu, Wentan Huang

**Affiliations:** Department of Nuclear Medicine, People’s Hospital of Guangxi Zhuang Autonomous Region, Nanning, Guangxi Zhuang Autonomous Region, China

**Keywords:** Graves’ hyperthyroidism, nomogram, prediction model, LASSO regression, radioiodine therapy

## Abstract

**Objective:**

To examine the factors influencing ^131^I-refractory Graves’ disease (GD) hyperthyroidism in patients, develop a nomogram prediction model, and conduct its validation.

**Methods:**

A total of 272 hyperthyroidism patients who received initial ^131^I treatment at our hospital from January 2021 to January 2022 were randomly selected. Patients were divided into refractory hyperthyroidism group (92 cases) and non-refractory hyperthyroidism group (180 cases) based on whether they were cured after one course of ^131^I treatment. They were randomly divided into a training group (n=190) and an internal validation group (n=82) in a 7:3 ratio. Multiple factors that might affect the efficacy of ^131^I treatment were collected, including 16 variables such as clinical characteristics, laboratory, and imaging examinations. LASSO regression was used for optimization and selection, and a multivariate logistic regression model was constructed to create a nomogram prediction model. The model’s discrimination, calibration, and clinical validity were evaluated using the receiver operating characteristic (ROC) curve, Hosmer-Lemeshow calibration curve, and decision curve analysis (DCA).

**Results:**

There were no statistically significant differences (P>0.05) in the comparison of the 16 variables between the training and validation groups. Following LASSO regression analysis, six predictive variables associated with ^131^I-refractory hyperthyroidism were identified: the duration of hyperthyroidism, nighttime sleep quality, the presence of Graves' ophthalmopathy (GO), the effective half-life of thyroid ^131^I, thyroid uptake ^99m^Tc value, and thyroid mass. The area under the ROC curve (AUC) for the risk of ^131^I refractory hyperthyroidism in the training group was 0.943 (95% CI: 0.909-0.977), and the AUC for the validation group was 0.926 (95% CI: 0.870-0.983). The Hosmer-Lemeshow calibration curve showed good fit (training group P=0.876; validation group P=0.202). DCA demonstrated that when the threshold probability for equal patients ranged from 0.04 to 0.86 in the training group and from 0.09 to 0.87 in the validation group, using the nomogram prediction model to predict the risk of refractory hyperthyroidism after ^131^I treatment was more beneficial.

**Conclusion:**

This study found that the duration of GD hyperthyroidism, nighttime sleep quality, GO, effective half-life of thyroid ^131^I, thyroid uptake ^99m^Tc value, and thyroid mass are independent influencing factors of ^131^I refractoriness. A risk prediction model including these six factors was established. This model provides guidance for the diagnosis and treatment decisions of ^131^I refractory GD hyperthyroidism, offers a quantitative tool for clinical assessment of ^131^I efficacy, and aids in personalized treatment decisions, reducing the burden of ineffective or inefficient treatments.

## 1 Introduction

Graves’ disease (GD) represents the predominant etiology of hyperthyroidism, accounting for approximately 80%-85% of cases (1). This condition is a prevalent autoimmune disorder impacting the endocrine system, characterized by the aberrant production of antibodies by the immune system, which target the thyroid gland and result in excessive secretion of thyroid hormones. The incidence of GD is associated with genetic predisposition and adverse social determinants. In recent years, there has been a noted increase in the prevalence of GD, which may be attributed to the rising pressures of contemporary social life. Persistent social stressors, such as work, financial challenges, and social media, can dysregulate the hypothalamic-pituitary-adrenal (HPA) axis and elevate cortisol levels, thereby promoting neuroinflammation and altering neural circuits, including amygdala hyperactivity and prefrontal cortex dysfunction (2). The principal therapeutic strategies for managing GD encompass three primary modalities: antithyroid drugs (ATD), radioactive iodine (^131^I), and partial thyroidectomy. Among these, ^131^I has emerged as the preferred treatment option due to its efficacy, safety, simplicity, minimal side effects, and cost-effectiveness (3). Although ^131^I therapy is generally associated with positive outcomes, a significant proportion of patients experience suboptimal results, necessitating repeated treatments with ^131^I (4). According to literature statistics, between 8% and 50% of patients do not achieve successful treatment with a single dose and require re-treatment (5). This highlights the urgent need for the development of improved therapeutic strategies to optimize patient outcomes and reduce associated healthcare costs.

Research into the factors influencing treatment resistance in GD has gained traction, as understanding these determinants is crucial for improving therapeutic efficacy. Various studies have identified potential predictors of treatment resistance, including disease duration and presence of Graves’ ophthalmopathy (GO) may correlate with suboptimal responses to ^131^I therapy (6, 7). Addressing these factors can provide valuable insights into the underlying mechanisms of resistance, thereby informing more effective treatment protocols.

This study undertakes a retrospective analysis of clinical data from a single center, encompassing 272 patients who ^131^I therapy for GD, with a specific focus on identifying independent risk factors associated with treatment resistance. Utilizing robust statistical methodologies, including LASSO regression analysis, the research seeks to elucidate the interactions between various clinical characteristics and treatment outcomes (8–10). The primary objective is to develop a predictive model that accurately identifies patients at risk of treatment failure, thereby informing clinical decision-making and enhancing patient care (11–13).

## 2 Materials and methods

### 2.1 Study subjects

Conduct a retrospective analysis of clinical data from 272 patients diagnosed with hyperthyroidism who received initial treatment with ¹³¹I at our hospital between January 2021 and January 2022. Among these patients, 74 were newly diagnosed and had not undergone any prior treatment for hyperthyroidism, while the remaining 198 had a history of ATD treatment but were otherwise untreated with ¹³¹I. The inclusion criteria were as follows: (1) a diagnosis of GD with first-time ^131^I therapy; (2) prior ATD treatment for a minimum duration before ¹³¹I therapy, specifically methimazole for at least two weeks or propylthiouracil for at least four weeks, during which all patients exhibited elevated thyroid function; and (3) the ability to communicate independently, without any psychological or mental disorders. The exclusion criteria were: (1) GD accompanied by severe infiltrative exophthalmos or significant underlying ocular conditions such as severe myopia or glaucoma; (2) the presence of hyperfunctioning thyroid adenoma; (3) concurrent hyperthyroid heart disease or other significant cardiac conditions, including heart failure; (4) a history of thyroid surgery; (5) the presence of malignant tumors or mental illnesses. This study received approval from the Ethics Committee of the People’s Hospital of Guangxi Zhuang Autonomous Region (approval number: KY-GZR-2025-035).

### 2.2 Pre-treatment preparation

Before receiving ^131^I treatment, patients had to refrain from iodine-containing medications and other substances that could affect ^131^I uptake for at least 2 weeks. The following examinations were required before treatment: (1) Laboratory tests: measuring serum levels of FT3, FT4, TSH, TgAb, TPOAb, and TRAb. (2) Thyroid iodine uptake rate determination: using ^131^I provided by Nanning Atomic High-throughput Isotope Co., Ltd. to measure thyroid iodine uptake rates. (3) Thyroid static imaging: using ^99m^Tc sodium pertechnetate provided by Nanning Atomic High-throughput Isotope Co., Ltd. to perform static imaging of the thyroid to measure thyroid weight. Thyroid iodine uptake rates at 3 and 24 hours were assessed through iodine uptake rate determination. The formula for calculating thyroid volume via thyroid radionuclide planar imaging is as follows: Thyroid volume (cm³) = average height of the two lobes (cm) × anterior projection area of the two lobes (cm²) × K, where K is a constant ranging from 0.23 to 0.32, depending on imaging conditions. The dosage of ^131^I was determined using the formula: dosage = [planned dose (MBq/g) × thyroid weight (g)]/maximum thyroid iodine uptake rate. The planned dose of ^131^I was set between 2.59 and 4.44 MBq/g (70 to 120μCi/g). Prior to treatment, informed consent was obtained, and patients were apprised of the necessary precautions related to ^131^I treatment.

### 2.3 Diagnostic criteria and data preprocessing

The effectiveness of ^131^I therapy for GD, assessed 3–6 months post-treatment, is classified as follows (14): Euthyroidism is identified by the absence of symptoms or signs of Graves’ hyperthyroidism (GH) and normal serum levels of free triiodothyronine (FT3), free thyroxine (FT4), and thyroid stimulating hormone (TSH). A diagnosis of hypothyroidism is made if a patient presents with symptoms or signs of hypothyroidism, or even in their absence, accompanied by subnormal FT3 and FT4 levels and elevated TSH levels. Partial remission is defined by the alleviation of GH symptoms, partial resolution of signs, and a reduction in serum FT3 and FT4 levels, although these do not return to normal. No response is characterized by either no significant improvement or a worsening of hyperthyroidism symptoms and signs, with no reduction in serum FT3 and FT4 levels. These outcomes are evaluated based on the impact of a single course of ^131^I therapy. Euthyroidism and hypothyroidism are considered indicators of non-refractory hyperthyroidism, whereas partial remission, no response, and relapse are indicative of refractory hyperthyroidism. GO eye signs are classified as follows: no eye signs; mild eye signs (characterized by eyeball protrusion of 19–20 mm, or accompanied by mild symptoms such as photophobia, foreign body sensation, tearing, and other minor ocular discomforts, with minimal impact on daily activities); and moderate eye signs (characterized by eyeball protrusion of 21–23 mm, or accompanied by ocular discomfort symptoms with a moderate impact on daily activities). Thyroid nodules are categorized as either absent or present. Nighttime sleep quality is assessed as follows: a duration of ≥7 hours is deemed good sleep, while less than 7 hours is considered poor sleep. The dosage of ^131^I therapy administered per gram of thyroid tissue is categorized as follows: a low dose ranges from 70-90 µCi/g, and a high dose ranges from 91-120 µCi/g.

### 2.4 Data collection

Data collected within one week prior to ^131^I therapy encompasses the following: ① General information, including age, gender, duration of hyperthyroidism, nighttime sleep status, GO eye signs, and the administered dose of ^131^I per gram of thyroid tissue; ② Laboratory test data, which involves fasting venous blood tests for thyroid peroxidase antibodies (TPOAb), thyrotropin receptor antibodies (TRAb), FT3, and FT4; ③ Examination data, comprising thyroid color ultrasound, thyroid function assessment to determine the 3-hour and 24-hour radioactive iodine uptake rates (RAIU-3h and RAIU-24h), and the effective half-life (RAIU-1/2) of ^131^I; and dual-head single-photon emission computed tomography (SPECT) for technetium-99m (^99m^Tc) thyroid imaging, which measures the ^99m^Tc uptake value and thyroid mass data.

### 2.5 Statistical methods

All statistical analyses were conducted using R version 4.2.3 and Python version 3.11.4 on the collected dataset. For inter-group comparisons, continuous data with normal distribution were presented as mean ± standard deviation and analyzed using t-tests, whereas non-normally distributed continuous data were represented as median (interquartile range) with Mann-Whitney U tests. Categorical data were expressed as relative frequencies and analyzed using the Chi-square test. Missing data for TPOAB and TRAB in certain patients were imputed using random forest methods. The cohort of 272 patients with GD was categorized into a refractory hyperthyroidism group (n=92) and a non-refractory hyperthyroidism group (n=180). These groups were further randomly divided into a training set (n=190) and an internal validation set (n=82) in a 7:3 ratio. The occurrence of refractory hyperthyroidism following ^131^I therapy served as the outcome variable. Independent risk factors were identified through LASSO regression analysis, and a nomogram prediction model was constructed using multivariate logistic regression analysis. The AUC assessed the prediction model’s ability to discriminate, with a range from 0.5 to 1.0, where values near 1.0 suggest superior predictive performance (15). The Hosmer-Lemeshow calibration curve assessed how well the prediction model fit the data, and the DCA curve evaluated its clinical validity (16, 17). A P value below 0.05 was deemed statistically significant.

## 3 Results

### 3.1 General information

In a cohort of 272 patients diagnosed with GD, 63 were male (23.16%) and 209 were female (76.84%), with a mean age of 28.75 ± 45 years. Of these patients, 92 (33.82%) exhibited refractory outcomes, whereas 180 (66.18%) were classified as non-refractory. No statistically significant differences were observed between the training group (n=190) and the internal validation group (n=82) regarding variables such as gender, age, duration of hyperthyroidism, nighttime sleep conditions, GO eye signs, presence of thyroid nodules, administered dose of ^131^I per gram of thyroid tissue, levels of TPOAb, TRAb, FT3, FT4, RAIU3h, RAIU24h, RAIU1/2, thyroid uptake of ^99m^Tc, and thyroid mass (P>0.05), as detailed in **Table 1**.

**Table 1:** Comparison of general data between training group and internal validation group patients.

Variable	Total (n=272)	Training set (n=190)	Internal validation set (n=82)	P-value
Age	38.00 [28.75;45.00]	38.00 [30.00;44.75]	39.00 [27.00;47.00]	0.694
Duration	12.00 [2.00;42.00]	12.00 [2.00;57.00]	6.00 [3.00;36.00]	0.229
FT3	25.70 [15.56;35.42]	25.03 [15.41;34.21]	28.13 [16.96;38.47]	0.279
FT4	59.53 [46.07;68.14]	56.78 [44.27;67.28]	62.78 [50.02;69.92]	0.089
TPOAB	345.80 [69.78;927.00]	339.80 [57.27;927.00]	423.55 [93.78;938.03]	0.767
TRAB	19.90 [10.35;33.70]	19.90 [10.75;32.00]	20.05 [8.50;35.95]	0.659
RAIU_3H	75.30 [57.58;87.00]	75.20 [56.20;86.43]	77.25 [61.67;87.00]	0.494
RAIU_24H	87.00 [78.07;94.42]	86.25 [76.93;93.15]	89.50 [80.08;96.00]	0.094
RAIU_Half_time	5.20 [4.70;5.90]	5.20 [4.73;5.90]	5.25 [4.60;5.97]	0.611
Technetium_uptake_ratio	37.40 [24.28;68.28]	36.00 [24.10;67.90]	41.15 [25.08;71.67]	0.630
Weight_of_thyroid_gland	52.45 [37.50;76.75]	56.05 [36.90;80.70]	48.75 [39.05;60.53]	0.136
Gender				0.637
Male	63 (23.16%)	42 (22.11%)	21 (25.61%)	
Female	209 (76.84%)	148 (77.89%)	61 (74.39%)	
Nighttime sleep quality				0.817
Good	168 (61.76%)	116 (61.05%)	52 (63.41%)	
Poor	104 (38.24%)	74 (38.95%)	30 (36.59%)	
Thyroid nodules				0.490
No	207 (76.10%)	141 (74.21%)	66 (80.49%)	
Yes	57 (20.96%)	42 (22.11%)	15 (18.29%)	
Eye_sign				0.774
No	208 (76.47%)	143 (75.26%)	65 (79.27%)	
Mild	38 (13.97%)	28 (14.74%)	10 (12.20%)	
Moderate	26 (9.56%)	19 (10.00%)	7 (8.54%)	
Dose per gram				0.762
Small doses (70–90 uCi/g)	158 (58.09%)	112 (58.95%)	46 (56.10%)	
Large doses (91–120 uCi/g)	114 (41.91%)	78 (41.05%)	36 (43.90%)	
Refractory hyperthyroidism				0.622
No	180 (66.18%)	128 (67.37%)	52 (63.41%)	
Yes	92 (33.82%)	62 (32.63%)	30 (36.59%)	

### 3.2 Variable selection

**Figure 1:**
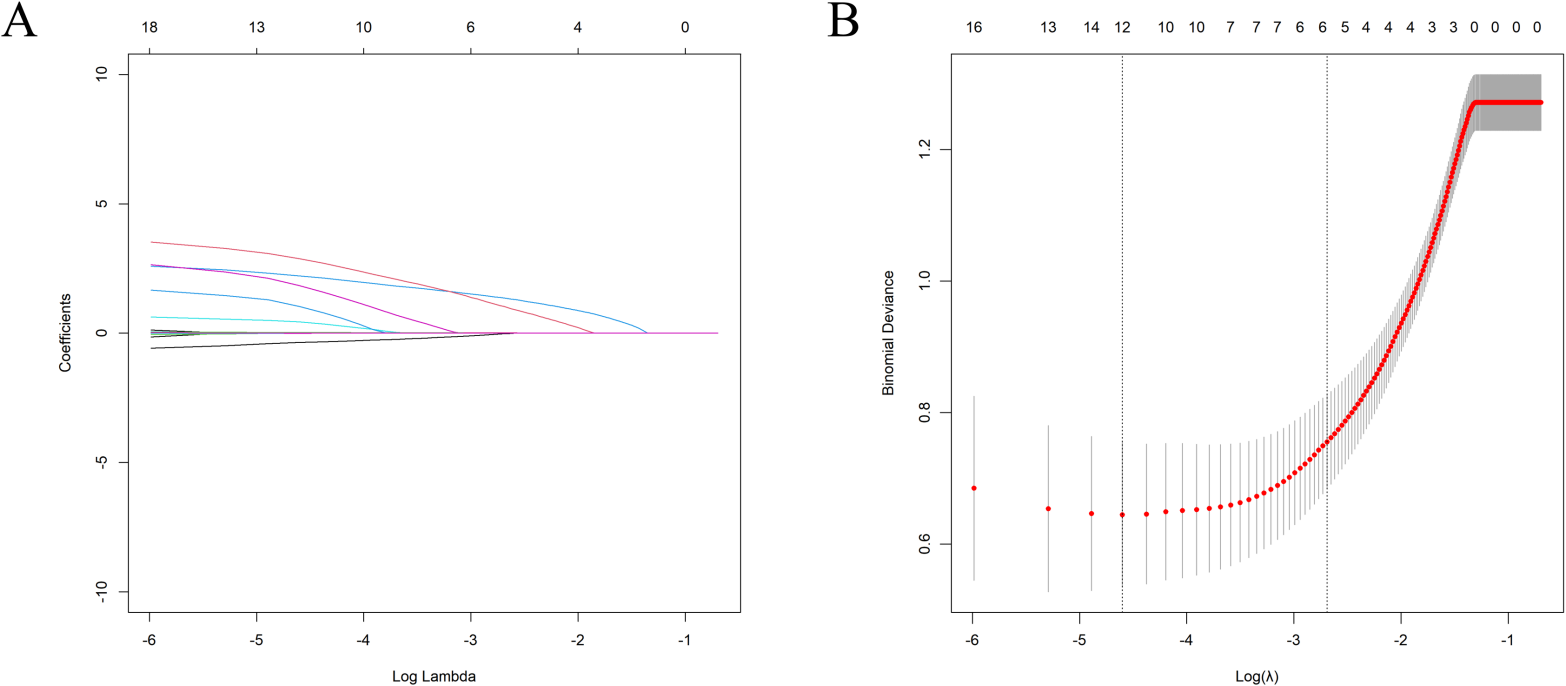
LASSO regression model for selecting predictive variables; **(A)** displays the coefficient curve of 16 clinical features, while **(B)** illustrates optimal variable selection via 10-fold cross-validation. A coefficient profile plot was generated in relation to the logarithmic sequence of λ. A vertical line was drawn at the selected value where the optimal λ yielded six nonzero coefficients.

Through the application of LASSO regression analysis, a total of 16 variables were optimized and selected, with the optimal value determined by the minimum 10-fold cross-validation error within one standard error (1SE) (refer to **Figure 1**). The λ corresponding to the minimum standard error of distance was identified as 0.067, leading to the selection of six predictive variables with non-zero coefficients. These variables include disease duration, GO eye signs, nighttime sleep conditions, RAIU1/2, thyroid uptake of ^99m^Tc, and thyroid mass.

### 3.3 Building predictive models

**Figure 2:**
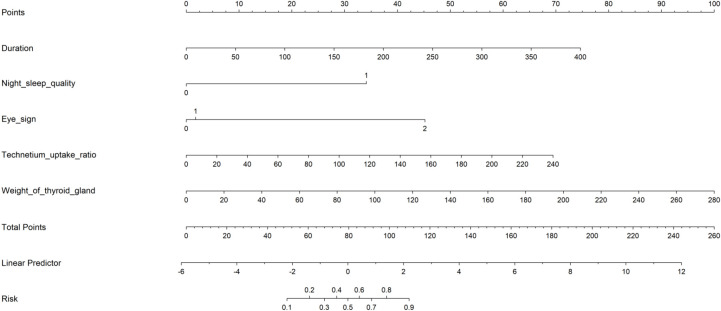
Nomogram predicting refractory outcome risk in GD patients treated with ^131^I therapy. To utilize the nomogram, locate the position of each variable on its respective axis, then draw a line to the “points” axis to ascertain the number of points for each variable. Sum the points from all variables, and subsequently draw a line from the total points axis to determine the probability of refractory hyperthyroidism following ^131^I therapy.

Multi-Factor Logistic Regression Analysis: This study employs a multi-factor logistic regression model to predict the refractory status of GD patients following their initial ^131^I therapy. The dependent variable is defined as the treatment outcome, categorized as either non-refractory (coded as 0) or refractory (coded as 1). Independent variables were selected using LASSO regression and include the duration of hyperthyroidism (measured in months), GO eye signs (coded as none=0, mild=1, moderate=2), and nighttime sleep quality (coded as good=0, poor=1). Additional independent variables are the RAIU1/2 (measured in days), thyroid uptake of ^99m^Tc (measured value), and thyroid mass (measured in grams). These six factors are utilized to develop a predictive model for treatment outcomes. The logistic regression analysis results are depicted in a straightforward visual nomogram (**Table 2**, **Figure 2**), illustrating that the total score is calculated by summing the scores derived from the univariate scoring scale for the six variables: duration of disease, GO eye signs, nighttime sleep quality, RAIU1/2, thyroid uptake of ^99m^Tc, and thyroid mass.

**Table 2:** Multivariate logistic regression analysis of the risk of refractory treatment in GD patients undergoing ^131^I therapy.

Variable	Coefficient	P-value	OR value	OR (95% CI)
Duration of hyperthyroidism	0.016	0.001	1.016	1.007-1.026
Nighttime sleep condition	2.747	0.000	15.595	5.617-51.113
RAIU1/2	-0.415	0.183	0.66	0.346-1.192
Thyroid uptake of ^99m^Tc	0.02	0.017	1.023	1.005-1.038
Thyroid mass	0.014	0.127	1.014	0.998-1.033
Eye_sign				
Mild	0.311	0.642	1.365	0.361-5.117
Moderate	3.506	0.00	33.314	6.075-296.559

### 3.4 Validation of the predictive model

**Figure 3:**
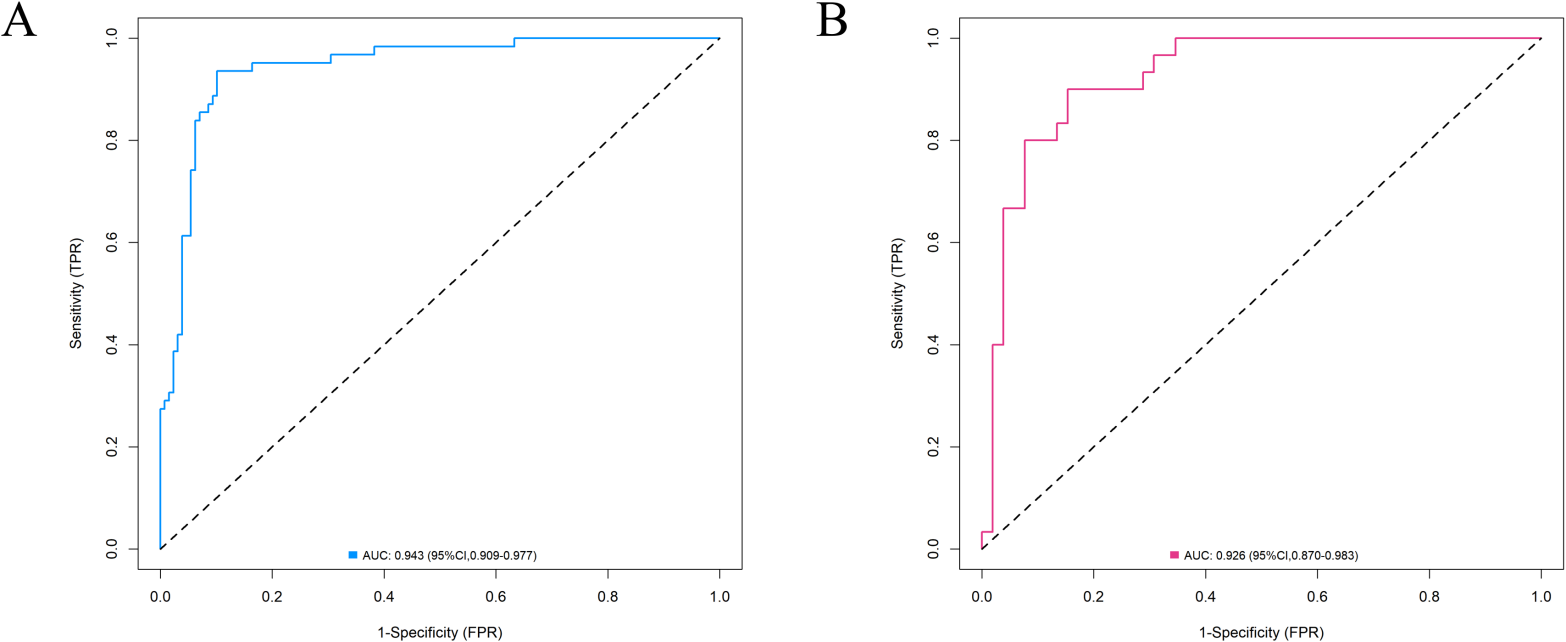
ROC curve of the prediction model for ^131^I-refractory hyperthyroidism [**(A)** training group, **(B)** internal validation group]. The AUC for the training group was 0.943 (95% CI: 0.909-0.977), and the AUC for the validation group was 0.926 (95% CI: 0.870-0.983).

The predictive models for the training group and the internal validation group were evaluated for accuracy using the ROC curve. The results showed that the AUC for the training group was 0.943 (95% CI: 0.909-0.977), and the AUC for the validation group was 0.926 (95% CI: 0.870-0.983), indicating high accuracy (see **Figure 3**).

**Figure 4:**
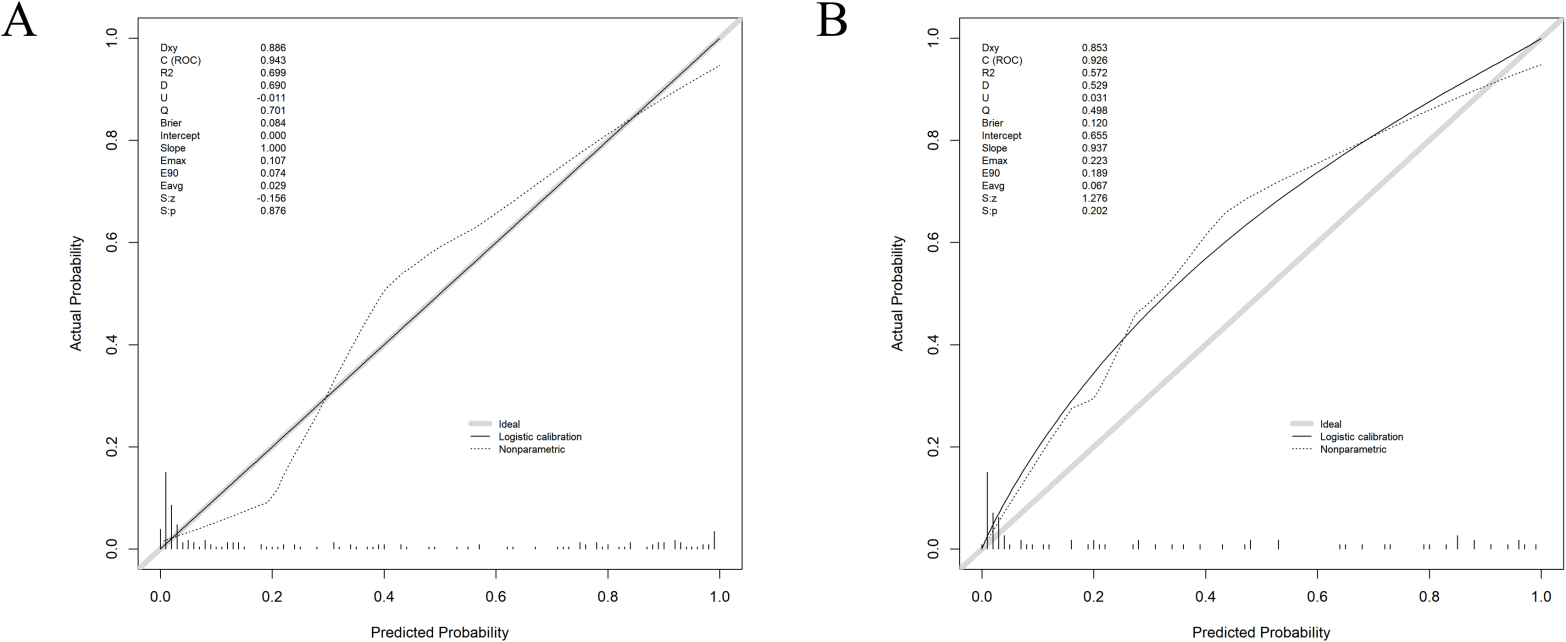
Hosmer-Lemeshow calibration curve of the prediction model [**(A)** training group, **(B)** internal validation group]. The Emax for the training and internal validation cohorts were 0.107 and 0.223, respectively. Similarly, the Eavg for these groups were 0.029 and 0.067, respectively (P=0.876, 0.202).

The Hosmer-Lemeshow calibration curve was employed to assess the goodness-of-fit, revealing that the maximum deviation (Emax) for the training and internal validation cohorts were 0.107 and 0.223, respectively. Similarly, the minimum deviation (Eavg) for these groups were 0.029 and 0.067, respectively (P=0.876, 0.202). All results successfully met the criteria for goodness-of-fit validation (see **Figure 4**).

**Figure 5:**
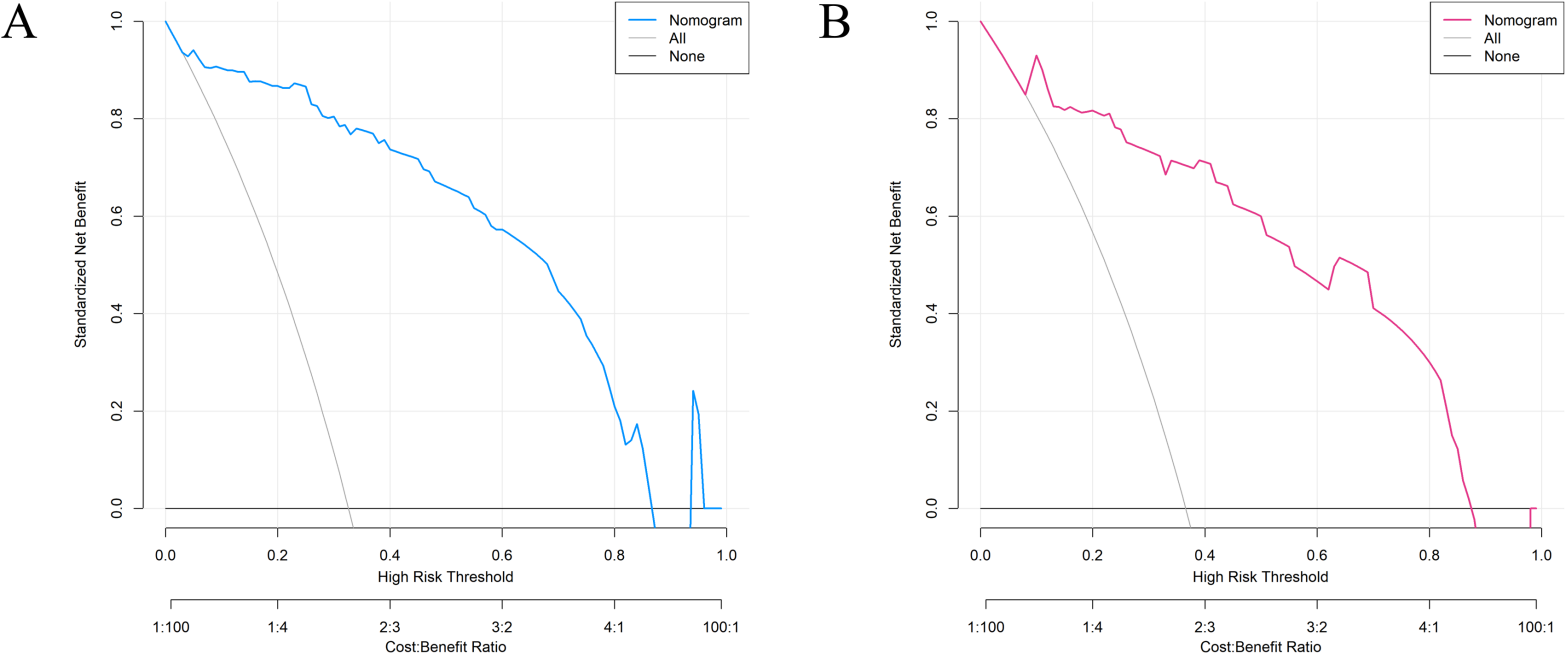
DCA curve of the prediction model. The threshold probability for equal patients ranged from 0.04 to 0.86 in the training group **(A)** and from 0.09 to 0.87 in the internal validation group **(B)**, employing the nomogram prediction model to assess the risk of refractory hyperthyroidism post-^131^I treatment was more advantageous.

The DCA curve was used for clinical effectiveness evaluation (see **Figure 5**), and the results indicated that when the threshold probability for equal patients ranged from 0.04 to 0.86 in the training group and from 0.09 to 0.87 in the internal validation group, employing the nomogram prediction model to assess the risk of refractory hyperthyroidism post-^131^I treatment was more advantageous. For example, assuming a high-risk threshold probability of 0.6 is selected, diagnosing patients with refractory hyperthyroidism and intervening yields a net benefit of 0.28, meaning that 28% of patients can benefit without harming the interests of others.

## 4 Discussion

This study undertook a retrospective analysis of 16 clinical data points from 272 patients diagnosed with GD, who initially received ^131^I therapy at our institution between January 2021 and January 2022. The objective was to identify factors influencing the efficacy of ^131^I therapy and to develop a risk prediction nomogram. Utilizing LASSO regression and multivariate logistic regression analysis, the study identified the duration of hyperthyroidism, nighttime sleep quality, GO eye signs, RAIU1/2, thyroid ^99m^Tc uptake values, and thyroid mass as independent risk factors for refractory hyperthyroidism post-^131^I therapy. The nomogram constructed from these variables exhibited strong predictive capability and calibration in both the training and internal validation cohorts, with the AUC recorded at 0.943 and 0.926, respectively. The DCA indicated that the prediction model provides practical benefits when the threshold probability ranges from 0.04 to 0.86 in the training group and from 0.09 to 0.87 in the internal validation group, thereby offering a scientific foundation for the individualized treatment of patients with GD.

The methodology utilized in this study is distinguished by its comprehensive approach, incorporating extensive data collection and rigorous statistical analysis. The clinical data encompass a diverse array of patient demographics, laboratory results, and treatment protocols, facilitating a nuanced understanding of the factors contributing to treatment resistance. By partitioning the patient population into training and validation cohorts, the study enhances the reliability and generalizability of its findings, thereby reinforcing the validity of the developed predictive model.

In summary, this study addresses a critical gap in the current literature by examining the risk factors associated with treatment resistance in patients with GD undergoing radioiodine therapy. The results are anticipated to deepen the understanding of the disease’s complexities and contribute to the formulation of personalized treatment strategies designed to improve patient outcomes. Future research could expand on these findings by investigating additional variables that may affect treatment efficacy and resistance, thereby facilitating further advancements in the management of GD.

The duration of hyperthyroidism, identified as the primary independent risk factor in this study, aligns with findings from previous research. Patients experiencing a prolonged duration of the illness may undergo alterations in thyroid tissue structure due to extended exposure to elevated thyroid hormone levels, resulting in reduced sensitivity to ^131^I therapy (18). Prior studies have demonstrated that variables such as illness duration and gender influence the effectiveness of oral antithyroid drug treatments (19–22). This study corroborates these findings by confirming that the duration of illness significantly affects the efficacy of ^131^I therapy. For patients with an extended duration of illness, it may be necessary to adjust the ^131^I dosage or consider combination therapy strategies to enhance treatment outcomes.

This study represents the inaugural investigation to incorporate nighttime sleep quality into the analysis of risk factors associated with refractory hyperthyroidism treated with ^131^I therapy. The findings indicate a significant correlation between poor sleep quality and suboptimal treatment outcomes. This discovery holds substantial implications for clinical practice, given the intricate relationship between sleep quality and the regulation of endocrine function (23, 24). Sleep disturbances may activate the HPA axis, leading to increased cortisol secretion, which indirectly amplifies the peripheral effects of thyroid hormones. This exacerbation complicates the management of hyperthyroid symptoms and adversely affects the efficacy of ^131^I therapy. Consequently, it is imperative in clinical settings to evaluate the sleep status of patients with hyperthyroidism and to provide appropriate guidance on sleep hygiene. Such measures may enhance the effectiveness of RAI.

GO, a common complication of hyperthyroidism, is shown in this study to be more prevalent among patients exhibiting positive ocular signs of GO. Specifically, individuals with pronounced ocular manifestations of GO (odds ratio [OR] = 33.314) demonstrate a higher likelihood of recurrence following treatment, which may be attributed to a more active autoimmune response (25). Some studies have suggested that the worsening of GO symptoms post-^131^I therapy could be linked to a temporary surge in TRAb release (26–28). Our findings indicate that patients with GD hyperthyroidism and positive GO ocular signs, when treated with standard doses of ^131^I, do not achieve optimal outcomes and may require either multiple or higher doses of ^131^I for effective management. Nevertheless, ocular signs of GO can significantly improve following ^131^I therapy, and the autoimmune response may be substantially suppressed post-treatment. This suggests that the presence of GO ocular signs could adversely affect treatment efficacy. Consequently, it can be deduced that GO eye manifestations are indicative of an active systemic autoimmune response. This particular immune response may confer a degree of radioresistance to thyroid cells, thereby necessitating higher doses of ^131^I to overcome this resistance and achieve optimal therapeutic outcomes. However, this hypothesis warrants further investigation for confirmation. Studies have reported alterations in cytokine levels, such as interleukin-2 (IL-2) and interleukin-6 (IL-6), in the serum of patients following ^131^I therapy (29, 30). These changes are closely associated with improvements in cardiac function among hyperthyroid patients, underscoring the significant role of immune factors in the management of hyperthyroidism. Therefore, for patients exhibiting mild to moderate GO eye signs, more personalized treatment regimens may be required, potentially involving increased ^131^I dosages to enhance treatment efficacy.

RAIU1/2 serves as an indicator of the metabolic rate of radioactive iodine within thyroid tissue. This study identifies RAIU1/2 as an independent risk factor influencing treatment outcomes, aligning with existing research on the effectiveness of low-dose ^131^I therapy for GD. Empirical evidence suggests that thyroid RAIU1/2 (OR=1.169) is a significant determinant of therapeutic efficacy, with prolonged RAIU1/2 times being associated with an increased risk of hypothyroidism. An extended RAIU1/2 signifies a longer retention of RAI in the thyroid, potentially affecting the therapeutic action of RAI on thyroid tissue (31, 32). Consequently, a shorter RAIU1/2 time corresponds to reduced retention of RAI in the thyroid, thereby diminishing its therapeutic efficacy. Therefore, when devising treatment protocols, it is imperative to consider the patient’s thyroid RAIU1/2 to optimize individualized therapeutic strategies.

In the context of diagnosing and evaluating the efficacy of hyperthyroidism treatments, ROI ratio in thyroid ^99m^Tc imaging emerges as a crucial predictor (33). This aligns with the findings of the present study, which indicate that thyroid ^99m^Tc uptake values serve as significant prognostic indicators for the outcomes of ^131^I therapy. Qi et al. utilized the ROI ratio from thyroid ^99m^Tc imaging to determine individualized ^131^I dosages for hyperthyroidism treatment, demonstrating that this approach is both straightforward and practical, with notable prognostic value (34). Both ^99m^TcO_4_^-^ and ^131^I are isotopes absorbed by the thyroid gland; however, ^99m^TcO_4_^-^ is absorbed through adsorption without undergoing organification, whereas ^131^I is absorbed and participates in organification metabolism (35, 36). The uptake of ^131^I by the thyroid is subject to various influencing factors, including prior administration of ATDs, consumption of iodine-rich foods, and the precision of measurement instruments, which can compromise the authenticity and stability of RAIU measurements. Consequently, the metabolic activity of ^99m^TcO_4_^-^ in the thyroid may provide a more accurate reflection of the gland’s metabolic capacity compared to RAIU. In this context, the metabolic intensity of ^99m^TcO_4_^-^ in the thyroid may provide a more precise indication of the gland’s metabolic capacity compared to the RAIU test. This observation also elucidates why RAIU measurements at 3 hours and 24 hours post-administration were not considered independent risk factors in this investigation. During thyroid imaging with ^99m^Tc, the ^99m^TcO_4_^-^ is absorbed by the sodium/iodide symporter (NIS) located on the membrane of thyroid follicular cells and subsequently transported into the cells. An elevated uptake of ^99m^TcO_4_^-^ by the thyroid correlates with enhanced NIS activity, which in turn reflects increased synthesis and secretion of thyroid hormones, thereby elevating the risk of recurrence following treatment. The findings of this study suggest that the metabolic activity observed in ^99m^TcO_4_^-^ imaging is inversely related to the sensitivity to ^131^I therapy, with higher thyroid ^99m^TcO_4_^-^ uptake values indicating a requirement for increased dosages of ^131^I.

This study corroborates the notion that thyroid mass serves as an independent determinant of the efficacy of ^131^I therapy. Patients presenting with larger thyroid masses exhibited suboptimal treatment outcomes, aligning with existing literature (32). This phenomenon may be attributed to the potential for uneven radiation dose distribution within larger thyroid masses, resulting in inadequate destruction of certain follicles and necessitating increased ^131^I doses to achieve a satisfactory therapeutic effect. Consequently, it is imperative to consider administering a higher dose of ^131^I in patients with larger thyroid masses to enhance treatment efficacy. These findings underscore the importance of conducting ^99m^Tc radionuclide thyroid imaging prior to ^131^I therapy to ascertain thyroid ^99m^Tc uptake values and assess thyroid mass. Such information is instrumental in predicting treatment outcomes and facilitating the development of individualized treatment plans.

In this study, the variable of the dose of ^131^I therapy per gram of thyroid tissue was not included as an independent risk factor; however, the effectiveness of ^131^I therapy remains closely associated with this parameter. According to the 2018 European Thyroid Association Guideline the formula for calculating the ^131^I dose is as follows: ^131^I treatment dose (µCi) = planned dose per gram of thyroid tissue (70-120 µCi) × thyroid mass × 100/maximum RAIU at 24 hours (5). While thyroid mass and maximum RAIU can be accurately measured through testing, the planned dose per gram of thyroid tissue presents a broad range of options, indicating significant uncertainty in its selection, which is critical for prognosis. Clinicians often take into account factors such as the patient’s thyroid size, tissue hardness, age, and duration of illness, resulting in noticeable individual practices and preferences. This study classified the planned dose per gram of thyroid tissue into two categories: small doses (70–90 uCi/g) and large doses (91–120 uCi/g), considering it as a variable potentially affecting outcomes. However, the findings indicated that this variable does not independently influence the treatment outcomes for refractory GD managed with ^131^I therapy. Consequently, the impact of varying planned doses per gram of thyroid tissue on the study outcomes was negated. Therefore, given the customary practices and preferences of thyroid medical practitioners at this institution regarding planned doses per gram of thyroid tissue, investigating other independent factors influencing the treatment of refractory GD with ^131^I therapy holds practical significance.

This study developed a nomogram prediction model for refractory hyperthyroidism treated with ^131^I therapy, utilizing six independent risk factors. The model construction adhered rigorously to statistical methodologies, employing LASSO regression for variable selection and multivariate logistic regression to identify independent risk factors, culminating in the creation of a visual nomogram. This approach is extensively utilized in medical research (37–39). We applied a range of statistical techniques to assess the performance of the prediction model. Firstly, the model exhibited strong discriminative capability in both the training and validation cohorts, with AUC values of 0.943 and 0.926, respectively. Secondly, the Hosmer-Lemeshow calibration curve demonstrated that the predicted probabilities closely aligned with the actual outcomes, with P-values of 0.976 and 0.202 for the training and validation cohorts, respectively, indicating no statistically significant difference. The Hosmer-Lemeshow test serves as a crucial technique for evaluating model calibration, where a P-value exceeding 0.05 suggests an adequate model fit (40). Furthermore, DCA demonstrated that when the threshold probability ranges from 0.04 to 0.86 in the training group and from 0.09 to 0.87 in the internal validation group, employing this model to predict the risk of refractory GD treated with ^131^I therapy yields substantial clinical benefits. Collectively, these statistical evaluation outcomes affirm the superior quality and practical utility of this model, surpassing numerous previously reported predictive models and underscoring its high accuracy.

In comparison to existing research, this study is pioneering in its comprehensive examination of multiple clinical characteristics, laboratory tests, and imaging indicators that may affect the efficacy of ^131^I therapy. Furthermore, we employed a combination of LASSO regression and multivariate logistic regression methods to enhance the scientific rigor of variable selection and ensure the reliability of our findings. LASSO regression, recognized for its efficacy in variable selection and regularization, reduces model complexity by introducing a penalty term, thereby mitigating overfitting and enhancing the model’s generalizability. Additionally, the nomogram we developed is both intuitive and user-friendly, which facilitates its application in clinical settings. We also undertook rigorous internal validation of the model to confirm its stability and reliability. Nonetheless, this study is not without limitations. As a retrospective analysis, it is susceptible to selection and information biases. The designation of retrospective studies often suggests lower research quality, a notion that warrants careful interpretation. Secondly, the relatively limited sample size may compromise the external validity of the findings. Despite conducting internal validation, the absence of external validation remains a significant limitation. Furthermore, certain clinical data, such as nighttime sleep quality, were predominantly derived from patient self-reports, lacking objective measurement tools, which may introduce recall bias. Retrospective studies are inherently constrained by the quality and completeness of medical records, and the potential influence of unrecorded confounding factors cannot be excluded (41). Additionally, this study was conducted at a single center, and the results may be affected by regional characteristics and hospital-specific factors. Epidemiological methods also present certain practical limitations, including issues related to diagnostic accuracy, population estimation, and migration.

Building upon the findings and limitations identified in this study, future research could be directed toward several key areas. Firstly, there is a need for multi-center, large-sample, prospective studies to further validate the reliability and applicability of our results. Secondly, future investigations should explore additional potential influencing factors, particularly at the molecular and genetic levels, such as examining the relationship between thyroid autoantibodies and the outcomes of RAI. Some studies have suggested that TRAb is not associated with initial cure rates and the incidence of hypothyroidism, which aligns with our findings (34). Thirdly, advancements in the construction of predictive models should be pursued, including the application of machine learning algorithms to enhance their predictive performance. Fourthly, research should focus on developing individualized treatment strategies for high-risk patients, such as adjusting ^131^I dosages and exploring combination drug therapies. Lastly, long-term follow-up studies are essential to evaluate the predictive value of the models for long-term outcomes.

## 5 Conclusion

We conducted a systematic analysis of the factors influencing refractory GD treated with ^131^I and developed a nomogram with robust predictive capabilities. Our findings reveal that the duration of hyperthyroidism, quality of nighttime sleep, presence of GO eye signs, RAIU1/2, thyroid ^99m^Tc uptake values, and thyroid mass are independent risk factors impacting the efficacy of ^131^I therapy. These results provide a scientific foundation for clinicians, facilitating risk stratification and personalized treatment decision-making. The nomogram we developed is user-friendly, intuitive, and shows promising potential for clinical application. Primarily, this model assists clinicians in identifying high-risk patients for ^131^I, enabling the implementation of more proactive intervention strategies. Furthermore, the model’s predictive outcomes can guide clinicians in tailoring individualized treatment plans, such as adjusting ^131^I dosages and incorporating additional therapies, thereby enhancing the overall effectiveness of ^131^I therapy. Additionally, the model can improve doctor-patient communication by helping patients comprehend their risk status, thereby increasing treatment adherence. In the future, our research should focus on external validation and optimization of the model to further improve its predictive performance and clinical application value.

## Data Availability

The original contributions presented in the study are included in the article/supplementary material. Further inquiries can be directed to the corresponding authors.
